# Volunteering across contexts: comparing attitudes toward volunteering with prisoners and people with mental illness

**DOI:** 10.3389/fpubh.2024.1432181

**Published:** 2024-10-28

**Authors:** Lara Dá Mesquita, Jaime Oliveira, Mariana Pinto da Costa

**Affiliations:** ^1^Institute of Biomedical Sciences Abel Salazar, University of Porto, Porto, Portugal; ^2^School of Medicine, Life and Health Sciences Research Institute (ICVS), University of Minho, Braga, Portugal; ^3^Center for Health Technology and Services Research (CINTESIS.UA), Department of Education and Psychology, University of Aveiro, Aveiro, Portugal; ^4^Institute of Psychiatry, Psychology and Neuroscience, King’s College London, London, United Kingdom

**Keywords:** attitudes, mental illness, prisons, inmates, volunteering, volunteers, qualitative study Portugal

## Abstract

**Introduction:**

Volunteering represents an opportunity for social transformation and social cohesion. Portugal is one of the European countries with fewer volunteering initiatives. Generally, society distances itself from individuals with mental illness and prison inmates, therefore, stigma becomes one of the barriers to social reintegration. However, volunteering can be a beneficial intervention helping individuals in their reintegration.

**Objectives:**

This study aims to compare the differences and similarities in the attitudes of volunteers toward volunteering with people with mental illness and prisoners.

**Methods:**

A supplementary qualitative secondary analysis was conducted using transcripts from 39 semi-structured individual interviews with volunteers regarding support of inmates in prison and two focus groups with volunteers regarding support of people with mental illness. Data analysis was conducted through an inductive thematic analysis.

**Results:**

Four themes emerged from the analysis: ‘Volunteer motivation and characteristics’, ‘Volunteer’s role’, ‘Volunteering relationship and its impact’, and ‘Challenges faced by volunteers’. There were several similarities between the perspectives toward volunteering in prisons and in mental health care, including the need for specific training in the area and the positive attitudes and behaviors of both groups of volunteers toward volunteering with the individuals supported. The differences were related to the characteristics necessary to be a volunteer, the activities carried out with the individuals supported and the difficulties faced by volunteers.

**Conclusion:**

These findings show overall positive attitudes toward volunteering in mental health and in prisons.

## Introduction

Volunteering is a pro-social action, without external impositions, driven by the will and personal motivations of social agents (1). This selfless endeavor yields benefits for others (2–4) and significantly contributes to social transformations, fostering social cohesion, and bridging gaps in community services (4, 5). The impact of volunteering extends to various aspects, including the reinforcement of solidarity, enhancement of overall well-being, and leading to a longer and healthier life (1, 5, 6). Moreover, it enriches the cultural and relational dimension of those who engage in it (2–4). Practiced in diverse contexts, volunteering encompasses different target groups and activities, varying according to the served population. Volunteering can take a formal or informal approach, with the former structured within organizations or associations (2, 4) and the latter being more spontaneous, occurring outside organizational contexts and directly undertaken by individuals, such as helping someone in crossing the street (4, 7, 8). The motivations for volunteering are rooted in internal or external factors (6, 9), influenced by elements like self-awareness and development, personal values, religion, social approval, and the desire to contribute to the improvement of social well-being and, gaining exposure to different realities.

Volunteering is a global phenomenon that occurs in various cultural and social contexts, subject to different legal frameworks (4, 10). According to the Portuguese legislation ‘Volunteering is an activity inherent to the exercise of citizenship that translates into a solidary relationship with others, participating in a free and organized manner in addressing the problems affecting society as a whole’ (11). Despite the increasing visibility of volunteering in the media and public policies, Portugal continues to have a small number of volunteering initiatives and ranks among the countries with the lowest volunteering rates in Europe (8). The volunteering rate in Portugal stood at 11.5% in 2012, decreasing to 7.8% in 2018 (12, 13).

This decline in volunteering rates contrasts with the higher rates of mental illness in Portugal (14%) compared with the global prevalence of mental illness (8.2%) (14), and high rates of imprisonment (15).

Globally, around 165 million people worldwide are affected by mental illness annually (16). Portugal stands out in Europe as the second country with a high prevalence of mental illness, representing 11.8% of the diseases in Portugal (16). Despite the disparities between the societal willingness to help people with mental illness and the social tendency to distance from them, both volunteers and healthcare professionals consider it beneficial for individuals with mental illness to be the focus of volunteering efforts (9, 17). Various studies have demonstrated the positive effects of volunteering, benefiting both individuals receiving support and the volunteers themselves (18). The positive outcomes for individuals include improvements in recovery, well-being, and satisfaction, leading them to feel valued, more integrated, and appreciated (6). Additionally, volunteering contributes to combating social isolation and stigma, which, thereby facilitating social adaptation and cohesion (9). Mental health volunteering is a multifaceted phenomenon that can range from a friendly and informal approach to a more therapeutic and formal intervention, whether active (more structured) or passive (19, 20). The essence of this volunteering is to provide social support to individuals by offering informal companionship and relieving the workload of healthcare professionals (9). Mental health volunteers offer support, hope, attentive listening, and encouragement to individuals, as well as activities to help them acquire social skills and autonomy, facilitating their recovery and subsequent social integration (17, 21). Maintaining a non-judgmental stance is crucial for volunteers (21).

Worldwide, there are 11.5 million inmates, with 93% being male and 7% female (22). In Portugal the number of inmates increased from 10,807 in 2008, to 11,704 in 2021 (23). Although volunteering in prison is not widely spread in Portugal, there was an increase in the number of volunteers (82%) and the number of supported inmates (51%) in four correctional facilities from 2005 to 2007 (24). Prison volunteering spans various areas, including the development of social and personal skills, cultural, artistic, educational, and training activities, improvement of prison facilities, relaxation activities, promotion of healthy lifestyles, and community connection (25). The goal of prison volunteering is to reduce social isolation and stigma, empower inmates with new skills both inside and outside prisons, promote reintegration, and enhance the well-being of inmates (26–28). The impact of these visits, although subject to variations based on their consistency and regularity, results in positive outcomes such as promoting positive thoughts and future perspectives among inmates, reducing the risk of recidivism and inappropriate behaviors during the incarceration period (26, 27, 29). Visits can be considered a form of informal control (30) and a way to facilitate the implementation and maintenance of programs in prison (31). These interactions provide a mechanism through which volunteers can monitor and influence inmates to adopt positive behaviors, (32) thereby reducing instances of misconduct (30). This corrective potential (29) can contribute to a safer and more controlled prison environment (30), while also strengthening the bond with volunteers. In addition to directly impacting inmates, visits play a crucial role in facilitating access to educational and rehabilitation programs within prisons and provide continuous monitoring and evaluation of inmates’ progress (32). This allows for adjustments to programs according to inmates’ individual needs and specific circumstances (31). These visits also contribute to a sense of normalcy and connection to the outside world, which can motivate inmates to engage positively in rehabilitation efforts and adhere to program requirements (33). Importantly, these voluntary visits are more easily implemented compared to other types of programs and are more economically accessible compared to other options (30).

Despite the different contexts, both individuals with mental illness and prison inmates experience significant social isolation and stigma, which impede their reintegration into society. Volunteers in both settings face unique challenges and opportunities in supporting these marginalized populations. Understanding the similarities and differences in volunteers’ experiences and attitudes toward volunteering with these two groups is crucial for developing effective volunteer programs and policies. The aim of this study is to compare the differences and similarities in the attitudes of volunteers toward volunteering with people with mental illness and prison inmates. Therefore, this study aims to address this research question: “What are the differences and similarities in the attitudes of volunteers toward volunteering with prison inmates and individuals with mental illness?”

## Methodology

### Study design

A supplementary qualitative secondary analysis was conducted (34–36). A more detailed methodological description has been published elsewhere (18).

### Data collection

Transcripts of semi-structured interviews on volunteering in prison establishments were used (27) conducted with 39 volunteer individuals affiliated with 14 organizations (from the cities of Lisbon, Coimbra and Porto) that promote prison volunteer activities in Portugal. Transcripts from focus groups on mental health volunteering with 12 volunteers were used, all connected to volunteer-promoting organizations based in the city of Porto (18).

### Sample characterization

The sample consisted of a total of 51 participants. The age range varied from 21 to 76 years (*M* = 49.96, SD = 16.21). The professions of the volunteers were categorized according to the Portuguese Censos classification of professions (37). Table 1 provides the details of the sociodemographic data of participants.

**Table 1:** Sociodemographic data of volunteers.

		Mental health volunteering		Prison volunteering	
Gender	Female	*n* = 9 (75%)		*n* = 24 (61.5%)	
	Male	*n* = 3 (25%)		*n* = 15 (38.5%)	
Age		[21, 66]	*M* = 38.4 *DP* = 14.5	[26, 76]	*M* = 53.3 *DP* = 15.2
Profession		Specialists in intellectual and scientific activities *n* = 7 Retired *n* = 2 Administrative personnel *n* = 1 Workers in personal services, protection, security and sales *n* = 1 Unskilled workers *n* = 1		Representatives of the legislative and executive bodies, leaders, directors, and executive managers *n* = 4 Specialists in intellectual and scientific activities *n* = 15 Retired *n* = 7 Technicians and intermediate-level professions *n* = 7 Administrative personnel *n* = 2 Unskilled workers *n* = 2 Unemployed *n* = 2	

### Thematic analysis

The transcripts were analyzed using Braun and Clarke’s thematic analysis (38), facilitated by QRS International NVivo 14 software (39), using an inductive approach (38).

In alignment with Braun and Clarke’s methodology, the analysis followed specific steps. Initially, the first author conducted a thorough reading of the transcripts to establish familiarity with the data. This was followed by multiple readings to identify initial codes. These codes were then discussed and refined in collaboration with the second author to ensure consistency and to incorporate different perspectives. The coding process was iterative, involving regular meetings between all the authors to discuss emerging themes and resolve any discrepancies. Throughout this process, the themes were refined and redefined according to the research question in order to avoid duplications and redundant themes.

## Results

Four themes emerged from the analysis (Table 2). The theme “Motivation and volunteer characteristics” addresses the reasons volunteers engage in volunteering within prison or mental health settings, ranging from solidarity to the influence of contact with other volunteers. It also encompasses personal characteristics that are relevant to these volunteering contexts, such as neutrality and commitment. The theme about the “Role of the volunteer” explores the functions and responsibilities performed by volunteers within each volunteering context. Some of the key functions include combating social isolation and promoting social reintegration. The theme about the “Volunteering relationship and its impact” examines the relationship between volunteers and inmates or individuals with mental illness, and the impact this relationship has on both volunteers and the individuals involved. This theme includes the development of emotional bonds, initial uncertainties and apprehensions, and personal growth. In the theme of the “Challenges faced by volunteers,” the discussion revolves around the difficulties and obstacles volunteers encounter when working with inmates and individuals with mental illness. This may include issues such as emotional management, interpersonal conflicts, and stigma dismantling.

**Table 2:** Themes and subthemes.

	Mental health volunteering	Prison volunteering
Motivation and volunteer characteristics	Solidarity and willingness to help Influence of contact with other volunteers Flexibility and neutrality of the volunteer Volunteer commitment	
	Necessary patience of the volunteer Anyone can be a volunteer	Necessary maintaining some distance Specific capabilities for being a volunteer
Role of the volunteer	Combatting social isolation Promoting self-esteem Supporting with social reintegration	
	Monitoring mental health changes	Discretion and avoidance of legal discussions
Volunteering relationship and its impact	Uncertainties and apprehensions in the initial interactions Close and empathetic relationship between volunteers and those supported Greater receptivity toward volunteers compared to others Personal growth of the volunteer Relativization of volunteers’ problems	
	Learning about mental illness	Familiarization with the prison environment
Challenges faced by volunteers	Observing significant results of volunteer support Deconstructing stigma and raising awareness in society	
	Difficulties in dealing with people with mental illness Shame and abandonment of people with mental illness	Confrontation with the prison environment as violent Focus on the crime committed by the inmate

**Figure 1:**
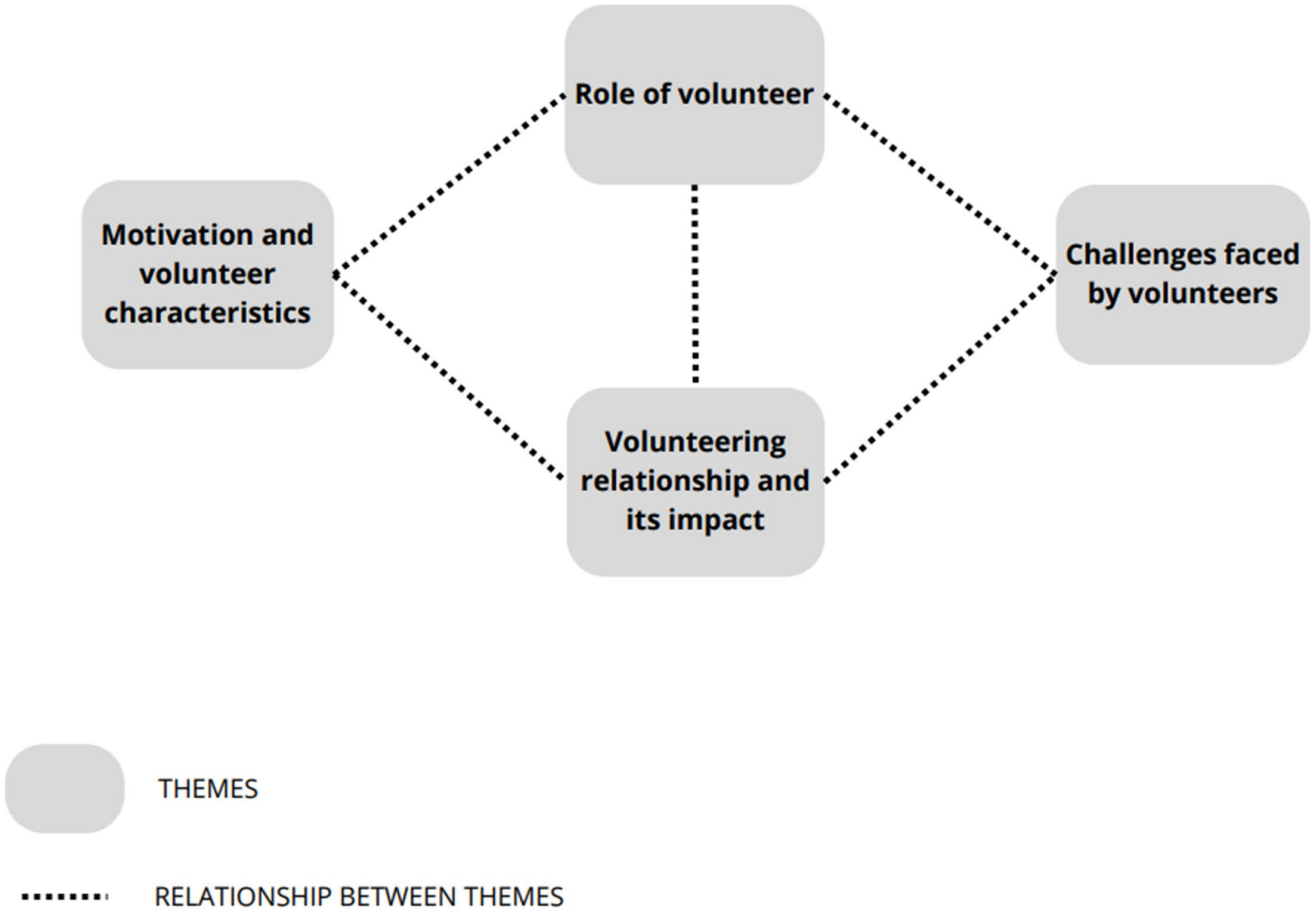
Relationships between themes.

There were mostly similarities found, although some differences were also observed. Relationships between themes are represented in Figure 1.

### Motivations and volunteer characteristics

#### Solidarity and willingness to help

For both settings (mental health care and prisons) it was stated that motivations for volunteering were driven by a desire to help, solidarity, and a willingness to participate in society. Both groups also expressed their desire to provide support to individuals, improve aspects of their lives, and collaborate in creating a more positive and just social environment.

*“[…] I just like happy people around me […] Because we like to see a more beautiful country […] happier, with less cruelty, less humiliation.”* (Focus Group of Mental Health Volunteers 01, Volunteer 03)

*“It’s an instinct, I think it’s an instinct for us to help, isn’t it?”* (Focus Group of Mental Health Volunteers 01, Volunteer 03)

*“I’ve always felt that we should find a way to participate in society and support people, and I’ve always had a tendency to look at the people around us.”* (Interview with Prison Volunteers, Volunteer 03)

*“[…] I think it was something like that, this need to want to improve people’s lives a little bit, even if it’s just a little.”* (Interview with Prison Volunteers, Volunteer 37)

#### Influence of contact with other volunteers

Both groups of volunteers reported that the exchange of experiences and interaction with other volunteers influenced their desire to participate in volunteering. Some highlighted personal reasons, such as family legacy, as a motivation to get involved. They expressed deep admiration for more experienced volunteers and felt capable of performing the proposed activities.

*“A friend once told me, I went to volunteer […] for a very personal reason because my godmother was a volunteer in a group […]We then spread the word, and there are many people saying, ‘I want to go with you, I want give it a try […]”* (Focus Group of Mental Health Volunteers 02, Volunteer 06)

*“I joined the [volunteering] program because I had one or two friends who did this kind of work and that I admired for doing this kind of work, and I thought ‘maybe I could do this kind of work’.”* (Interview with Prison Volunteers, Volunteer 03)

#### Flexibility and neutrality of the volunteer

Both groups of volunteers mentioned that volunteers should exhibit flexibility and neutrality. They perceived themselves as adaptable to different situations and individuals, so that individuals do not feel treated differently. They also highlighted the importance to maintain a non-judgmental attitude.

*“[…] the volunteer should be an adaptable being and an adaptable professional as well, we should try to reconcile all our skills as much as possible.”* (Focus Group of Mental Health Volunteers 02, Volunteer 02)

*“[…] act with her a bit differently, with other kinds of care, which we wouldn’t have with the others […] and at the same time try to be careful so that she wouldn’t notice that we were treating her differently so that she wouldn’t feel different.”* (Focus Group of Mental Health Volunteers 01, Volunteer 01)

*“[…] we have to make a constant effort not to judge the other person in front of us. I think that is something that is essential, not allowing any judgment of people.”* (Interview with Prison Volunteers, Volunteer 36)

*“I always adapt the way I behave to the person […]”* (Interview with Prison Volunteers, Volunteer 20)

#### Volunteer commitment

The volunteering was seen by both groups as a serious commitment. Participants mentioned that it is important to be regular and continue volunteering, as there are relationships to maintain. They also pointed out that they avoided failing the commitment to prevent disappointment and a lack of trust in the individuals they support.

*“[…] regardless of all our problems, we have to be willing and say, okay, this is to be taken seriously […] I agree that volunteering once or being a volunteer is different, it’s not the same thing […]”* (Focus Group of Mental Health Volunteers 02, Volunteer 06)

*“Personally, I try to do my best not to miss it because I don’t like to miss commitments, and this is a commitment that always requires some continuity, regularity because they also wait for us, and if we do not go, we contribute to their disappointment, letdown, and we do not want that because the little trust and connection that we gradually build could end up being lost.”* (Prison Volunteers Interview, Volunteer 38)

*“[…] it’s not about doing it for a year and then stopping just because; there are relationships that are formed, and while for us time passes quickly and we have several distractions, for them, it doesn’t.”* (Interview with Prison Volunteers, Volunteer 39)

### Necessary patience of the volunteer VS necessary maintaining some distance

For mental health volunteers, patience and empathy were considered necessary because they dealt with different realities and understanding them could be difficult. On the other hand, prison volunteers believed that it was necessary and important to maintain some distance, being aware that they were only there to perform their role as volunteers, to converse and provide companionship.

*“[…] their world is different from ours, maybe it’s not even physiological for us to understand what that person is thinking at that moment, or what they are seeing, experiencing. So you need to have patience, I think, more than finding empathy, it’s patience […]”* (Focus Group of Mental Health Volunteers 01, Volunteer 01)

*“[…] I have always addressed them informally, but I don’t give them much leeway to address me informally. There are certain boundaries that we must ensure; we are not their best friends in the sense of being there to give them hugs. No, we are there to keep them company, to talk with them, and to help as much as possible, especially when they come outside.”* (Interview with Prison Volunteers, Volunteer 03)

#### Anyone can be a volunteer VS specific capabilities for being a volunteer

In terms of the necessary characteristics for volunteering, there were different views in the two groups of volunteers. Mental health volunteers asserted that anyone who wished to become a volunteer could do so as long as they had availability. In contrast, prison volunteers expressed the opposite, emphasising that specific abilities were required to be a volunteer. These necessary skills included maturity, sound judgment, attentiveness, regular attendance, and emotional detachment.

*“Everyone can embrace our cause, everyone can embrace volunteering, but then we, especially the group administrators, also have to be attentive to the behaviour of the volunteers.”* (Focus Group of Mental Health Volunteers 02, Volunteer 06)

*“[…] I think the opportunity should be extended to all the people who have the availability to do it […]”* (Focus Group of Mental Health Volunteers 01, Volunteer 04)

*“[…] one must have some attention, some maturity, some regular attendance, continuity, emotional distance, common sense, and not everyone has these characteristics […]”* (Interview with Prison Volunteers, Volunteer 38)

*“So, I think that perhaps not all people will have the personal characteristics to be able to avoid getting [too] involved and to be volunteers […]”* (Interview with Prison Volunteers, Volunteer 36)

### Role of the volunteer

#### Combatting social isolation

Both mental health volunteers and prison volunteers concurred that the volunteer’s role revolved around addressing social isolation. They also stated that offering companionship and maintaining an active interaction provided emotional benefits to the individuals receiving support, fostering a sense of comfort and dignification.

*“It is often about trying to break down that barrier between loneliness and the rest of the group, and the rest of almost all of society.”* (Focus Group of Mental Health Volunteers 02, Volunteer 05)

*“They gain companionship, and that’s also a good thing.”* (Focus Group of Mental Health Volunteers 01, Volunteer 02)

*“So, this is also a work of dignification; many of them haven’t received [visits from] people for years, even from family members. So there’s a work here of presence, of addressing their isolation and deep loneliness in many cases […]”* (Interview with Prison Volunteers, Volunteer 06)

*“This is very comforting for them […] Our presence is a feeling that fills that emptiness inside […] It’s the feeling that, to them, we are marked, and that we do them good, and that they feel good with us […]”* (Interview with Prison Volunteers, Volunteer 01)

#### Promoting self-esteem

Another commonality reported by the two groups of volunteers was the promotion of self-esteem in the individuals supported by the volunteers. Volunteers spoke about their efforts in assisting individuals to engage in self-care practices and fostering an improved self-perception. To promote this well-being, volunteers identified key facilitators, such as acknowledging the limitations of the individuals, dignifying their experiences, and expressing affection.

*“[…] I think that’s essentially it, helping people feel better about themselves.”* (Focus Group of Mental Health Volunteers 01, Volunteer 04)

*“[…] we have to have the ability to handle the situation, to understand the person’s limitations, in order to help them, to encourage them to take a little extra care of themselves.”* (Focus Group of Mental Health Volunteers 02, Volunteer 04)

*“Sometimes it’s simple things like literally bringing love to these people, and bringing love is showing these people that they matter. There are people who say ‘no one has ever looked me in the eyes like you do, with respect,’ and this is painful to hear, but we know that we can make a difference.”* (Interview with Prison Volunteers, Volunteer 31)

*“[…] often it’s also about boosting their self-esteem, to make them understand that they are also worthy of a life because, as I was saying, they are ostracized, they are heavily labelled, and many of them think that life is over, but life is not over.”* (Interview with Prison Volunteers, Volunteer 14)

#### Supporting with social reintegration

There was agreement among both groups of volunteers regarding their role in supporting individuals in their reintegration into society. Participants perceived their interaction with individuals as a form of training for social practices, providing a period for internalizing societal norms. Prison volunteers also noted that this guidance extended beyond the prison context.

*“Because for a person to be integrated into society, they need to be with people first, right? And the volunteer ends up being a way to practice being with different people they don’t know at all, and it can also be a help.”* (Focus Group of Mental Health Volunteers 01, Volunteer 01)

*“[…] integrating [them] makes the person feel like a member of a family, right? […] help them feel like they have a friend, a sister, a brother, a cousin […] it will also help them see that they are capable of contributing and doing something for society.”* (Focus Group of Mental Health Volunteers 02, Volunteer 05)

*“[…] so, this is the society we have, and they need to understand that these are our rules, and that it’s good for them to follow the rules that society imposes on them even if they don’t understand them. It’s about realizing that we can’t always act as isolated individuals; we live in society, and it’s a bit about teaching them how to live in society.”* (Interview with Prison Volunteers, Volunteer 31)

*“[…] not only inside the prison, as has already happened, but also outside from the perspective of guidance, guiding them towards a life outside, a social life, to readapt them to everyday life […] This support outside is very important and is either nonexistent or very limited, only for a few. Re-entering society is very difficult for them because they leave with a dark mark on their lives […]”* (Interview with Prison Volunteers, Volunteer 01)

#### Monitoring mental health changes VS discretion and avoidance of legal discussions

Mental health volunteers focused on monitoring changes in mental health, identifying behavioral changes in the individual, such as isolation, rebellion, and aggression. In contrast, prison volunteers underscored the significance of discretion and avoidance of legal discussions as important in their approach. The latter stated that they did not want to know the specific crimes committed by the individual to prevent additional judgments about the supported individual.

*“[…] being attentive to the behaviours of those who are more isolated, those who show signs of depression, those who are more irritable, who are more rebellious, aggressive, those who don’t come to us, and so we go to them. Always respecting their space.”* (Focus Group of Mental Health Volunteers 02, Volunteer 05)

*“[…] because it’s a difficult and heavy context, and then because it’s necessary to know how to make this emotional separation, and not everyone can do it. Common sense, the ability to listen, […] trying not to talk about their legal cases, having maturity, regular attendance, continuity, a sense of commitment […].”* (Interview with Prison Volunteers, Volunteer 39)

*“[…] we never ask, we do not want to know the person’s crime because the person is already there, serving their sentence, we don’t need to add another label on them.”* (Interview with Prison Volunteers, Volunteer 21)

### Volunteering relationship and its impact

#### Uncertainties and apprehensions in the initial interactions

Both groups of volunteers referred to uncertainties and apprehensions during their initial interactions. They experienced an initial shock when faced with [prisoners’] unsolvable or less straightforward problems compared to their own. They identified the need for training, and they expressed a lack of knowledge on how to handle certain situations or what to expect from these individuals. Participants said that these initial feelings gradually disappeared, giving rise to spontaneous interaction and a warm welcome from the individuals they supported.

*“[…] I had no idea how I should deal with her, of course I would try to make her feel better, but I could probably make her feel worse, and it wasn’t, it wasn’t my intention, I was trying to help, but since I do not know how to handle it, I could be doing the wrong thing, not out of malice but there is that risk, I think there is that risk.”* (Focus Group of Mental Health Volunteers 01, Volunteer 04)

*“And we see problems that have no solution. Or at least, not as straightforward as some of our problems, I think that was the thing that shocked me the most on the first visit.”* (Focus Group of Mental Health Volunteers 02, Volunteer 03)

*“[…] the first time I entered, I confess that I thought, ‘What am I going to find?*’” (Interview with Prison Volunteers, Volunteer 10)

*“At first, I entered the prison a bit scared. They played some pranks on me, left me alone in a cell full of inmates; it’s part of the initiation. But I quickly realized I was in a place where I felt very respected and very appreciated.”* (Interview with Prison Volunteers, Volunteer 14)

*“[…] since it was the first time I had set foot in a prison, I had never had contact with one, I was a bit scared, ‘I’m going to be a woman doing an activity alone with young men,’ but actually, from the beginning, everything was very spontaneous…”* (Interview with Prison Volunteers, Volunteer 32)

#### Close and empathetic relationship between volunteers and those supported

Participants from both groups concurred that they upheld a positive, close and empathetic relationship with the individuals they supported, devoid of prejudice. Some volunteers of mental health and prisons went as far as to describe their connection with those they supported as friends. They highlighted that boundaries were set as per the volunteers’ preferences, and some chose to use the individuals’ first names to foster a respectful approach.

*“I think it ultimately comes down to this, or rather, the limit is, as far as we want it, because maintaining a very close emotional relationship with some is a matter of empathy or not…”* (Focus Group of Mental Health Volunteers 02, Volunteer 06)

*“[…] they like us, we like them too, and we create an empathy, and we get along well, we don’t even know why, but there are cases where I think it’s necessary for us to have some knowledge of the person’s illness, of what can suddenly happen to that person because there are illnesses that can cause the person to change their personality, the way we talk to them, anything can trigger a reaction that’s not very good.”* (Focus Group of Mental Health Volunteers 02, Volunteer 05)

*“[…] there’s a phrase we like to use, which is, ‘We are the friend who arrives when everyone else leaves…’“*(Interview with Prison Volunteers, Volunteer 07)

*“[…] it’s a relationship of complicity; they end up getting to know us, and they are also free from any prejudice towards us. We are just people who are there to help, and that’s quite positive.”* (Interview with Prison Volunteers, Volunteer 10)

*“[…] with them it’s a close relationship; I make a point of addressing them by name and having them address me by my name as well, and they are just like us because that’s exactly what they are.”* (Interview with Prison Volunteers, Volunteer 15)

#### Greater receptivity toward volunteers compared to others

Both groups considered that the individuals they supported exhibited greater receptivity toward them in comparison to others. Participants noted that their status as impartial outsiders to the system made individuals feel more comfortable and willing to open up about their difficulties. They also emphasized their commitment to confidentiality, ensuring that any information shared by the individuals remained private.

*“We don’t accept an opinion from a family member or a friend as easily; perhaps if it’s someone who is not directly connected to us, they end up providing us with company, they end up talking a lot more, than perhaps…, there are things they tell the volunteer but not to their father…” (Focus Group of Mental Health Volunteers 01, Volunteer 02)*

*“[…] they also saw us as outsiders with whom they could exchange ideas with, vent a little, and talk about the difficulties they were facing…”* (Interview with Prison Volunteers, Volunteer 28)

*“[…] there is a great need on their side to talk because we do not belong to the prison system, so they know that what they tell us stays with us.”* (Interview with Prison Volunteers, Volunteer 35)

### Personal growth of the volunteer

The personal growth of the volunteer was a shared perspective among both groups. Participants described the volunteering experience as rewarding, emphasising that it contributed not only to their personal enrichment but also to other areas of their lives. They considered that they acquired distinctive skills and developed an awareness of the existence of different realities beyond their own context.

*“And I think volunteering really enriches us internally a lot.”* (Focus Group of Mental Health Volunteers 01, Volunteer 03)

*“[…] but ultimately, I found it rewarding because when they came to me, they wanted to know, I could see that they wanted to learn, even without being able to express themselves or speak, I could see it in their eyes that they wanted to touch what I was working with…it was indeed rewarding, and I would like to do it again and have these opportunities.”* (Focus Group of Mental Health Volunteers 02, Volunteer 07)

*“Now, on a human level, it certainly adds, of course, human knowledge, flexibility, a range of other things …, I learn from it as a person, and I imagine that this always adds value to who I am as a professional in my field, as a daughter, sister, woman. I think it adds immense value to every aspect of my life, without a doubt, because I realise that my reality is not the only one…. These experiences translate into real and distinctive skills for the volunteer and for any citizen.”* (Interview with Prison Volunteers, Volunteer 25)

#### Relativization of volunteers’ problems

Relativization by the volunteer was another shared perspective among the two groups. Participants regarded volunteering as a means to distance themselves from their personal issues. They considered that through interactions with the individuals they were supporting, their perspectives expanded and evolved, increasing their awareness of diverse realities, prompting them to relativize some of their own problems and deconstruct pre-existing beliefs.

*“I also think that there are several volunteers who go through a phase of depression, and almost all of them say that it’s very good to get out and help others, because perhaps they understand better the problems that others are going through, and for them, it’s even a way of overcoming, of not thinking about their problems and focusing on others.”* (Focus Group of Mental Health Volunteers 02, Volunteer 05)

*“[…] it provided different solutions for a problem that I might have thought my solution was the only one. So it was more about becoming aware that in mental health, and in volunteering, truly becoming aware of the difficulties and problems that exist, and also the solutions.”* (Focus Group of Mental Health Volunteers 02, Volunteer 06)

*“In fact, going to the prison changes our perspective on how we look at other people… everyone… it changes the perspective with the inmate, changes the way we see them as men. They’re no longer just a men who stole or killed. It’s already a person, a much more complex human being, with good things…”* (Interview with Prison Volunteers, Volunteer 14)

*“One of the main advantages is realizing that our problems are not as serious when we hear the stories of some of the inmates. So, this relativisation of our problems, the deconstruction of this reality, the reduction of prejudices, the ability to listen, the emotional capacity…”* (Interview with Prison Volunteers, Volunteer 39)

*“Benefit is waking me up to reality, drawing my attention to the context in which I live in is not the only one that exists, that there are other social, economic, spiritual, emotional, moral, and even religious contexts that are very different from mine. So it draws my attention to the fact that reality is more complex than the reality I experience every day.”* (Interview with Prison Volunteers, Volunteer 16)

#### Learning about mental illness VS familiarization with the prison environment

In terms of volunteers learning, differences emerged between the two volunteer groups. In mental health, volunteers focused on acquiring knowledge about mental illness, enabling them to better understand the human mind and its challenges. On the other hand, volunteers in prison settings prioritized learning about and familiarizing themselves with the prison environment. They depicted the prison environment as heavy and complex, reporting not knowing what this reality was like before engaging in volunteering.

*“[…] I think I would have a completely different understanding of the human mind from the moment I knew something about the problems the human mind can suffer, wouldn’t I?”* (Focus Group of Mental Health Volunteers 01, Volunteer 01)

*“[…] it’s a very special environment, very heavy, very tense, where there are many people the same age as my children, where there are people my age or older than me, and where there are people who are completely abandoned… and so, I did not know about this before joining.”* (Interview with Prison Volunteers, Volunteer 03)

### Challenges faced by volunteers

#### Observing significant results of volunteer support

For both groups of volunteers, observing significant and direct outcomes of the support they provided was a major challenge. Participants described as difficult maintaining the interest of individuals, clarifying the role of volunteers, gaining acceptance for assistance, meeting the needs of individuals, and achieving positive results.

*“[…] being able to reach the patient with a mental health condition and truly meet their basic needs, to help them understand, and also understanding if they saw my intentions, and that they, in the end, felt better…”* (Focus Group of Mental Health Volunteers 02, Volunteer 03)

*“[…] if we see that the other person is not happy with our help, it’s a bit frustrating for us. We are trying to help, and we do not see that we are helping; we are making the situation worse, and that is not very good for us.”* (Focus Group of Mental Health Volunteers 01, Volunteer 04)

*“I think it’s very difficult, to maintain their interest because they don’t see the direct results… maintaining these people’s interest and really showing them the purpose we serve in their lives, showing our role, I think that’s the big challenge.”* (Interview with Prison Volunteers, Volunteer 30)

*“The challenge is to be able to achieve positive results from situations that are initially negative.”* (Interview with Prison Volunteers, Volunteer 33)

### Deconstructing stigma and raising awareness in society

Deconstructing stigma and advocating for a shift in public opinion were challenges mentioned by both groups. Some volunteers reported that individuals with mental health issues were perceived as odd or akin to “rare animals” in terms of their way of thinking, sometimes even treated as subjects for study. Volunteers spoke of a social belief associating these individuals as careless. They emphasized that the most significant challenge was attempting to alert societal perception, making people aware that above all, these individuals were human beings deserving to be treated with dignity.

*“[…] interpreting the thoughts of people with mental illness is like treating them as if they were animals, as if they do not think 100% like us, right?”* (Focus Group of Mental Health Volunteers 01, Volunteer 03)

“*My biggest challenge is even before anything else, making the relevant people to understand that mental health and the homelessness are human beings who deserve all the dignity and not the kind of responses where people from community or therapeutic centres who ask them, ‘either this or the street, no other option,’ because the state has already spent too much money on you.”* (Focus Group of Mental Health Volunteers 02, Volunteer 01)

*“[…] another thing is society; they leave already with a label, and a criminal record doesn’t disappear overnight. Nowadays, everyone wants to check the criminal record, and when it shows up, society doesn’t accept them. And when it does accept them, we have seen some who were accepted, but later, when certain people in society find out that there’s a former prisoner, they make [their] life hell.”* (Interview with Prison Volunteers, Volunteer 34)

*“Usually, people imagine a scenario, everyone looking terrible, all degraded. And there, you find everything, there are many who are an example that in their day-to-day, they get dressed, they look neat, and there are people with degrees, lawyers, enforcement agents.”* (Interview with Prison Volunteers, Volunteer 02)

*“[…] I think many people go there, and they go with an almost a voyeuristic attitude, as if they were rare animals. They go to observe, but these people are perfectly normal, just like us. But they go there to conduct so many studies that sometimes they say, ‘Oh my God, everyone seems to want to study us, as if we are animals in a cage, and everyone comes here to ask questions.’“*(Interview with Prison Volunteers, Volunteer 15)

*“One thing I often say, which is that the rule of law is left at the prisons gates… So, in prisons, there hasn’t been possible to change and advance in humanisation because all parties represented in the Assembly of the Republic are afraid to move forward with any initiative because they believe they will be penalised in the elections […] The biggest challenge is exactly this: changing public opinion because the political power is sensitive.”* (Interview with Prison Volunteers, Volunteer 20)

### Difficulties in dealing with people with mental illness VS confrontation with the prison environment as violent

Regarding the challenges faced, differences were found in the two groups of volunteers. In mental health, volunteers reported having difficulties in understanding, approaching, and evaluating the mental state of the individuals they supported. Conversely, in prison settings, volunteers identified adapting to the complex and often violent prison environment as a challenge, given its circumstances different from the usual.

*“In other words, their world is different from ours, and perhaps it’s not even physiological for us to understand what that person is thinking at that moment…”* (Focus Group of Mental Health Volunteers 01, Volunteer 01)

*“When I dealt with people with mental disabilities, I was a bit confused about the way they approached me…”* (Focus Group of Mental Health Volunteers 02, Volunteer 07)

*“Many times, I don’t have a perception of whether the person is in their right mind…”* (Focus Group of Mental Health Volunteers 01, Volunteer 02)

*“The adaptation to the prison environment itself, I think, is a very big challenge…”* (Interview with Prison Volunteers, Volunteer 37)

*“I think everything in the prison context is a challenge, first because we are not psychologists, and second because we are dealing with people in circumstances completely different from our own.”* (Interview with Prison Volunteers, Volunteer 33)

### Shame and abandonment of people with mental illness VS focus on the crime committed by the inmate

Mental health volunteers spoke about the shame associated with caring for individuals with mental illness and that the tendency was to hide this fact or resort to institutionalizing them in psychiatric hospitals. In the prison context, volunteers highlighted that the focus was placed on the crime committed by the individual rather than on the individual themselves, with inmates being judged based on the crime they committed.

*“In the past, people were even ashamed when they had someone with disabilities at home […] They were ashamed, they would take them and put them in Conde Ferreira Hospital, and now it’s not Conde Ferreira, it’s Magalhães Lemos Hospital. And then they would even abandon them because they were ashamed that society knew they had mentally disabled people.* (Focus Group of Mental Health Volunteers 01, Volunteer 03)

*“Much focus is placed on the crime the person committed and not on the person themselves.”* (Interview with Prison Volunteers, Volunteer 16)

*“The worst was this one; it’s pretty clear. It was with a paedophile, you see, I could not get past it because I had a son, and they also had a son of the same age, and this paedophile would always ask us, ‘And the boys?’ and then they started showing us pictures of his godson who slept with him and gave him a gold chain, and that’s when we almost ran away from him because I find paedophilia a horrible disease, a horrible perversion, children at risk…”* (Interview with Prison Volunteers, Volunteer 14)

### Relationships between themes

The themes identified in this study demonstrate a complex interplay that reflects the intricate nature of volunteer experiences and their diverse roles (Figure 1). “Motivation and Volunteer Characteristics” influence the nature and quality of the “Volunteering Relationship and its Impact.” Volunteers driven by motivations such as solidarity and a willingness to help may develop strong, empathetic relationships with the individuals they support. For instance, one volunteer stated, “*I’ve always felt that we should find a way to participate in society and support people and I’ve always had a tendency to look at the people around us.*” This desire to contribute to society positively affected their interactions with those they assist, as illustrated by another volunteer who noted, “*It’s an instinct, I think it’s an instinct for us to help, is not it?*”

The “Volunteering Relationship and its Impact” also directly affects the “Role of the Volunteer.” A robust, empathetic relationship may enhance a volunteer’s ability to effectively combat social isolation, promote self-esteem, and support social reintegration. This is evidenced by volunteers who reported that building strong relationships with inmates or individuals with mental illness helped them provide more meaningful support. For example, a volunteer mentioned, “*We are people there to help and that’s it. It’s quite positive.*”

The specific “Role of the Volunteer” presents unique “Challenges Faced by Volunteers.” Volunteers in mental health settings reported difficulties in dealing with the complexities of mental illness. One volunteer stated, “*Many times I do not have a perception of whether the person is in their right mind*.” Meanwhile, prison volunteers faced challenges in adapting to the environment and maintaining emotional detachment, as reflected by a volunteer’s comment, “*The adaptation to the prison environment itself I think is a very significant challenge*.” These challenges require volunteers to develop new skills and coping strategies, influencing their overall effectiveness and experience.

Furthermore, the “Challenges Faced by Volunteers” impact their “Motivation and Volunteer Characteristics.” Volunteers who encounter significant difficulties without sufficient support may experience burnout and reduced motivation. One volunteer highlighted, “*I personally try to do my best not to miss because I do not like to miss commitments and this is a commitment that always requires some continuity, regularity.*”

Overall, the interconnections between these themes highlight the dynamic and multifaceted nature of volunteering. The motivations and characteristics of volunteers shape their relationships with those they support, which in turn affect their roles and the challenges they face. Addressing these challenges can further influence volunteers’ motivations and characteristics, creating a continuous cycle of impact and adaptation.

## Discussion

### Key findings and comparison with other literature

In this study, both groups of volunteers highlighted solidarity and the influence of contact with other volunteers as motivational factors. These motivations align with previous research, where volunteers reported altruism, a commitment to social causes, a sense of social responsibility, and influences from third-party recommendations or inspirations (6, 9, 18, 20, 26, 31, 40–44). However, in this study, mental health volunteers also mentioned the desire to gain recognition and social status as a motivation for volunteering. This aligns with findings from a systematic review that noted mental health volunteers seeking acknowledgement and social approval through their involvement in volunteering (6). In contrast, prison volunteers in this study cited motivations centred on personal growth and stepping out of their comfort zones, consistent with a systematic review that identified similar motivations, including a willingness to confront uncomfortable situations (44).

Regarding the profile of prison volunteers in this study, there was a predominance of female volunteers, a finding consistent with findings from a systematic review (26, 43). Additionally, volunteers in this study had an average age of 53.3 years and, in most cases, high levels of education. These characteristics align with those found in a systematic review, where prison volunteers were, on average, middle-aged and often had higher levels of education (42, 44). Regarding the characteristics of prison volunteers, this research identified the importance of attributes such as flexibility, neutrality, attentiveness, consistency, and the ability to maintain emotional distance. Similarly, a study in the United States noted that, volunteers in prison settings needed to demonstrate maturity, emotional stability, flexibility, the capacity to maintain emotional distance, and ability in monitoring manipulative behaviors (31).

In this study both groups of volunteers unveiled positive attitudes and behaviors toward volunteering with both studied populations. These findings, align with a systematic literature review that identified favorable attitudes and behaviors of volunteers toward volunteering with people with mental illness (40). Additionally, research conducted in Hong Kong concluded that both general volunteers and prison volunteers demonstrated more positive attitudes compared to individuals who did not engage in volunteer activities (26).

In this study, both mental health and prison volunteers reported experiencing feelings of fear and apprehension during their initial interactions with the supported individuals. This reaction aligns with findings from other studies, including a systematic review, where volunteers expressed feelings of fear and nervousness when engaging with individuals with mental illness (21, 40).

Regarding the dynamic between the volunteer and the inmate, this study identified that inmates placed greater trust in volunteers compared to other individuals, viewing them as external and impartial figures. This finding aligns with the results from studies in the Netherlands and in Portugal (24, 29, 45). According to a systematic review, volunteers perceived their role as positive for people with mental illness (40), similarly to the findings of the present study. The role of prison volunteers in this study involved combatting inmate isolation, promoting their self-esteem, and preparing them for social reintegration. These results were similar to those found in a systematic review, where the role of the volunteer encompassed providing support and guidance to inmates, acting as a link to the outside world, challenging stereotypes, and promoting healthy relationships (24, 31, 44).

This study underscores that volunteering yields substantial benefits for both the supported individuals and the volunteers. This is consistent with the findings from a systematic review (40). After their volunteering experience, both groups of volunteers expressed gratitude for personal growth across various dimensions of their lives. This growth was evident in the acquisition of distinct skills, expanded perspectives, and as knowledge of diverse realities that prompted a revaluation of their life and questioning of pre-existing beliefs. These benefits align with previous studies, highlight the acquisition of new skills and perspectives, relational and personal growth, broadening horizons, appreciation for specific aspects of life, feelings of completeness, fulfilment, satisfaction, and reward (6, 18–20, 24, 40, 42, 44, 46, 47). Furthermore, this study findings align with the results of two systematic reviews reporting volunteering as an opportunity for volunteers to confront and reassess biases toward people with mental illness (40, 43). As reported in a systematic review, volunteers in prison settings gain a greater awareness of the challenges faced by inmates, enabling them to overcome preconceived stereotypes (44).

In this study, mental health volunteers encountered challenges concerning understanding and interacting with the supported individuals. These difficulties align with observations in a systematic review, emphasising the intricacies volunteers confront in responding to and managing resistance from those they supported (40). Similarly, prison volunteers in this study faced challenges akin to those documented in a study in the United States, where volunteers grappled with difficulties in dealing with inmates, encompassing emotional aspects, and the process of overcoming and reassessing perspectives (31). Additionally, the prison volunteers participating in this study reported challenges associated with adapting to the often hostile and complex prison environment. This discomfort and dissatisfaction with the prison environment were also highlighted as challenges in a systematic review (44).

Finally, as highlighted by a systematic review, the effectiveness of volunteering in prison contexts fundamentally hinges upon volunteer training and supervision, the allocation of tasks appropriate to the context, and the fostering of a cooperative relationship between volunteers and correctional staff (39). However, in our study, we did not obtain any data regarding the training and supervision of volunteers.

### Strengths and limitations

This study represents the first comparison of attitudes toward volunteering in mental health volunteers and in prisons. The sample was diverse in terms of age, profession, and volunteer experience, allowing for a variety of perspectives.

However, this study has some limitations. First, the number of interviewees who discussed mental health volunteering was relatively small (12 participants) compared to those who were interviewed about prison volunteering (39 participants). This study findings might also be limited by the focus on a single geographic region in Portugal, which may constrain the generalizability of the results to broader populations of both mental health and prison volunteers.

Whilst this study methodology relies on self-reported attitudes and experiences of volunteers, there may be some degree of social desirability bias present. Additionally, the qualitative thematic analysis, while providing rich insights, is inherently interpretive.

### Implications of the results for practice and future research

This study reports differences in perceptions of volunteering in mental health and prison settings, stemming from the distinct nature of these areas of volunteering and the specific needs of these populations.

Firstly, it can assist in the development of high-quality training programs and personalized support tailored to the specific needs and challenges faced by the individuals being helped. This targeted approach not only may enhance volunteers’ satisfaction and effectiveness but may also have a positive impact on both programs and the individuals being assisted. Moreover, comprehending volunteers’ attitudes may influence recruitment strategies, which may impact in volunteers’ retention. Further understanding of social attitudes and perceptions can contribute to combating stigmas and stereotypes, thereby facilitating the rehabilitation and reintegration of these marginalized populations.

Future research could adopt a comprehensive methodological approach to enhance our understanding of volunteer attitudes and motivations. Such research could consider expanding the sample size, incorporating multiple geographic locations, and utilizing targeted recruitment strategies to ensure the representation of diverse volunteer demographics. Additionally, studies might benefit from triangulating qualitative data with other sources, such as observational data or behavioral measures, to obtain a more comprehensive picture of volunteer attitudes and motivations. Furthermore, intentionally investigating the potential intersections of such factors such as gender, ethnicity, age, or socioeconomic status might influence attitudes toward and the volunteer experience. This would provide a more nuanced understanding of how systemic disparities might manifest in contexts of social volunteering.

To enhance volunteering practice in these mental health and prison settings, some practical recommendations are proposed. First, promoting greater awareness of volunteering in these contexts can attract a larger pool of volunteers. Additionally, simplifying and streamlining recruitment procedures is advised to facilitate volunteer participation. Understanding the motivational patterns of volunteers is crucial, given that motivation significantly influences volunteer behaviors and their engagement duration. Further suggestions include providing specific volunteers training and implementing stricter supervision to ensure intervention quality. Innovative intervention strategies are proposed for supporting individuals in these settings, as well as improvements of the volunteering facilities. Establishing or strengthening support networks and community connections is considered essential for the assistance and the social reintegration of supported individuals. Moreover, emphasising the importance of changing public perception by involving society directly in volunteering reality and demonstrating transparency in initiatives is crucial for enhancing understanding and empathy. Educating society to perceive these individuals as human beings beyond mental illness or crimes committed, is vital to combat stigma effectively.

To address the knowledge gaps in volunteering in Portugal, several research areas are proposed. This includes conducting comprehensive assessments of existing volunteer programs to ascertain their effectiveness and pinpoint areas for improvement. Additionally, it is crucial to investigate the motivational patterns driving volunteering. The impact of volunteer visits on, both the supported individuals and the volunteers should be studied. Longitudinal studies are recommended to analyze the impact of volunteering over time. It is important to conduct research that considers the perspectives of individuals receiving volunteer support, the volunteers themselves, and society at large, thereby enhancing the understanding and optimization of the benefits and dynamics of volunteering.

## Conclusion

This study conducted a comparative analysis of the attitudes of volunteers toward volunteering with people with mental illness and prison inmates, with respect to their characteristics, motivations, the role of the volunteer, the volunteer-supportee relationship, its impact, and the challenges faced by volunteers.

The results revealed positive attitudes of volunteers toward volunteering with the supported individuals, with some relationships evolving into friendships. Both mental health and prison volunteers reported a positive impact on their lives, emphasising the importance of training and specific knowledge in their respective volunteering areas.

However, the study highlighted the need for specific characteristics and skills for volunteers in each context. Some mental health volunteers were motivated by a desire for social recognition, while prison volunteers were driven by personal growth. The approaches of both groups differed, with mental health volunteers adopting a flexible stance, while prison volunteers exhibited a more cautious and regulated approach. Activities and knowledge acquisitions varied between the two volunteering contexts. These findings also raise awareness of the importance of volunteering in reducing social stigma and promoting overall well-being for both supported individuals and volunteers.

For a more comprehensive understanding, future studies could benefit from a larger sample of mental health and prison volunteers.

## Data Availability

The transcripts analyzed are available from MP. Requests to access these datasets should be directed to mariana.pintodacosta@gmail.com.
