# Supplementary material for: Volunteering across contexts: comparing attitudes toward volunteering with prisoners and people with mental illness
Source: Front Public Health. 2024 Oct 28;12:1432181. doi: 10.3389/fpubh.2024.1432181 (PMC11550934; doi:10.3389/fpubh.2024.1432181)
Supplement: Supplementary file 1 [file Data_Sheet_1.docx]

## **Appendix 1 -** *Themes, subthemes and quotations*

|  | **Mental health volunteering** | **Prison volunteering** |
| --- | --- | --- |
| **Motivation and volunteer characteristics** | | |
| **Similarities** | **Solidarity and willingness to help** | |
|  | *"[...] I just like happy people around me [...] Because we like to see a more beautiful country [...] happier, with less cruelty, less humiliation."*  **(Focus Group of Mental Health Volunteers 01, Volunteer 03)**  *"It's an instinct, I think it's an instinct for us to help, isn't it?"*  **(Focus Group of Mental Health Volunteers 01, Volunteer 03)** | *"I've always felt that we should find a way to participate in society and support people, and I've always had a tendency to look at the people around us."*  **(Interview with Prison Volunteers, Volunteer 03)**  *"[...] I think it was something like that, this need to want to improve people's lives a little bit, even if it's just a little."*  **(Interview with Prison Volunteers, Volunteer 37)** |
|  | **Influence of contact with other volunteers** | |
|  | *"A friend once told me, I went to volunteer […] for a very personal reason because my godmother was a volunteer in a group [...]We then spread the word, and there are many people saying, ‘I want to go with you, I want to give it a try [...]"*  **(Focus Group of Mental Health Volunteers 02, Volunteer 06)** | *"I joined the [volunteering] program because I had one or two friends who did this kind of work and that I admired for doing this kind of work, and I thought 'maybe I could do this kind of work'."*  **(Interview with Prison Volunteers, Volunteer 03)** |
|  | **Flexibility and neutrality of the volunteer** | |
|  | *“"[...] the volunteer should be an adaptable being and an adaptable professional as well, we should try to reconcile all our skills as much as possible."*  **(Focus Group of Mental Health Volunteers 02, Volunteer 02)**  *"[...] act with her a bit differently, with other kinds of care, which we wouldn't have with the others [...] and at the same time try to be careful so that she wouldn’t notice that we were treating her differently so that she wouldn't feel different."*  **(Focus Group of Mental Health Volunteers 01, Volunteer 01)** | *"[...] we have to make a constant effort not to judge the other person in front of us. I think that is something that is essential, not allowing any judgment of people."*  **(Interview with Prison Volunteers, Volunteer 36)**  *"I always adapt the way I behave to the person [...]"*  **(Interview with Prison Volunteers, Volunteer 20)** |
|  | **Volunteer commitment** | |
|  | *"[...] regardless of all our problems, we have to be willing and say, okay, this is to be taken seriously [...] I agree that volunteering once or being a volunteer is different, it's not the same thing [...]"*  **(Focus Group of Mental Health Volunteers 02, Volunteer 06)** | *"Personally, I try my best not to miss it because I don't like to miss commitments, and this is a commitment that always requires some continuity, regularity because they also wait for us, and if we don't go, we contribute to their disappointment, let down, and we don't want that because the little trust and connection that we gradually build could end up being lost."*  **(Interview with Prison Volunteers, Volunteer 38)**  *"[...] it's not about doing it for a year and then stopping just because; there are relationships that are formed, and while for us time passes quickly and we have several distractions, for them, it doesn't."*  **(Interview with Prison Volunteers, Volunteer 39)** |
| **Differences** | **Necessary patience of the volunteer** | **Necessary maintaining some distance** |
|  | *" […] their world is different from ours, maybe it's not even physiological for us to understand what that person is thinking at that moment, or what they are seeing, experiencing. So, you need to have patience, I think, more than finding empathy, it's patience [...]"*  **(Focus Group of Mental Health Volunteers 01, Volunteer 01)** | *"[...] I have always addressed them informally, but I don't give them much leeway to address me informally. There are certain boundaries that we must ensure; we are not their best friends in the sense of being there to give them hugs. No, we are there to keep them company, to talk with them, and to help as much as possible, especially when they come outside."*  **(Interview with Prison Volunteers, Volunteer 03)** |
|  | **Anyone can be a volunteer** | **Specific capabilities for being a volunteer** |
|  | *"Everyone can embrace our cause, everyone can embrace volunteering, but then we, especially the group administrators, also have to be attentive to the behaviour of the volunteers."*  **(Focus Group of Mental Health Volunteers 02, Volunteer 06)**  *"[...] I think the opportunity should be extended to all the people who have the availability to do it [...]"*  **(Focus Group of Mental Health Volunteers 01, Volunteer 04)** | *"[...] one must have some attention, some maturity, some regular attendance, continuity, emotional distance, common sense, and not everyone has these characteristics [...]"*  **(Interview with Prison Volunteers, Volunteer 38)**  *"So, I think that perhaps not all people will have the personal characteristics to be able to avoid getting [too] involved and to be volunteers [...]"*  **(Interview with Prison Volunteers, Volunteer 36)** |
| **Role of the volunteer** | | |
| **Similarities** | **Combatting social isolation** | |
|  | *"It is often about trying to break down that barrier between loneliness and the rest of the group, and the rest of almost all of society."*  **(Focus Group of Mental Health Volunteers 02, Volunteer 05)**  *"They gain companionship, and that's also a good thing."*  **(Focus Group of Mental Health Volunteers 01, Volunteer 02)** | *"So, this is also a work of dignification; many of them haven't received [visits from] people for years, even from family members. So there's a work here of presence, of addressing their isolation and deep loneliness in many cases [...]"*  **(Interview with Prison Volunteers, Volunteer 06)**  *"This is very comforting for them [...] Our presence is a feeling that fills that emptiness inside [...] It's the feeling that, to them, we are marked, and that we do them good, and that they feel good with us [...]"*  **(Interview with Prison Volunteers, Volunteer 01)** |
|  | **Promoting self-esteem** | |
|  | *"[...] I think that's essentially it, helping people feel better about themselves."*  **(Focus Group of Mental Health Volunteers 01, Volunteer 04)**  *"[...] we have to have the ability to handle the situation, to understand the person's limitations, in order to help them, to encourage them to take a little extra care of themselves."*  **(Focus Group of Mental Health Volunteers 02, Volunteer 04)** | *"Sometimes it's simple things like literally bringing love to these people, and bringing love is showing these people that they matter. There are people who say 'no one has ever looked me in the eyes like you do, with respect,' and this is painful to hear, but we know that we can make a difference."*  **(Prison Volunteers Interview, Volunteer 31)**  *"[...] often it’s also about boosting their self-esteem, to make them understand that they are also worthy of a life because, as I was saying, they are ostracized, they are heavily labelled, and many of them think that life is over, but life is not over."*  **(Interview with Prison Volunteers, Volunteer 14)** |
|  | **Supporting with social reintegration** | |
|  | *"Because for a person to be integrated into society, they need to be with people first, right? And the volunteer ends up being a way to practice being with different people they don't know at all, and it can also be a help."*  **(Focus Group of Mental Health Volunteers 01, Volunteer 01)**  *"[...] integrating [them] makes the person feel like a member of a family, right? [...] help them feel like they have a friend, a sister, a brother, a cousin [...] it will also help them see that they are capable of contributing and doing something for society."*  **(Focus Group of Mental Health Volunteers 02, Volunteer 05)** | *"[...] so, this is the society we have, and they need to understand that these are our rules, and that it's good for them to follow the rules that society imposes on them, even if they don't understand them. It's about realizing that we can't always act as isolated individuals; we live in society, and it's a bit about teaching them how to live in society."*  **(Interview with Prison Volunteers, Volunteer 31)**  *"[...] not only inside the prison, as has already happened, but also outside from the perspective of guidance, guiding them towards a life outside, a social life, to readapt them to everyday life [...] This support outside is very important and is either nonexistent or very limited, only for a few. Re-entering society is very difficult for them because they leave with a dark mark on their lives [...]"*  **(Interview with Prison Volunteers, Volunteer 01)** |
| **Differences** | **Monitoring mental health changes** | **Discretion and avoidance of legal discussions** |
|  | *"[...] being attentive to the behaviours of those who are more isolated, those who show signs of depression, those who are more irritable, who are more rebellious, aggressive, those who don't come to us, and so we go to them. Always respecting their space."*  **(Focus Group of Mental Health Volunteers 02, Volunteer 05)** | *"[…] because it's a difficult and heavy context, and then because it's necessary to know how to make this emotional separation, and not everyone can do it. Common sense, the ability to listen, [...] trying not to talk about their legal cases, having maturity, regular attendance, continuity, a sense of commitment [...]."*  **(Interview with Prison Volunteers, Volunteer 39)**  *"[...] we never ask, we don't want to know the person's crime because the person is already there, serving their sentence, we don't need to add another label on them."*  **(Interview with Prison Volunteers, Volunteer 21)** |
| **Volunteering relationship and its impact** | | |
| **Similarities** | **Uncertainties and apprehensions in the initial interactions** | |
|  | *"And training, yes, I think that's important because I have no idea how to deal with these people..."*  **(Focus Group of Mental Health Volunteers 01, Volunteer 04)**  *"I think it's a very specific area, and perhaps there aren't many people trained for it. Because, we don't know how to handle most situations."*  **(Focus Group of Mental Health Volunteers 01, Volunteer 02)**  *"And we see problems that have no solution. Or at least, not as straightforward as some of our problems, I think that was the thing that shocked me the most on the first visit."*  **(Focus Group of Mental Health Volunteers 02, Volunteer 03)** | *"[...] the first time I entered, I confess that I thought, 'What am I going to find?'"*  **(Interview with Prison Volunteers, Volunteer 10)**  *"At first, I entered the prison a bit scared. They played some pranks on me, left me alone in a cell full of inmates; it's part of the initiation. But I quickly realized I was in a place where I felt very respected and very appreciated."*  **(Interview with Prison Volunteers, Volunteer 14)**  *"[...] since it was the first time I had set foot in a prison, I had never had contact with one, I was a bit scared, 'I'm going to be a woman doing an activity alone with young men,' but actually, from the beginning, everything was very spontaneous..."*  **(Interview with Prison Volunteers, Volunteer 32)** |
|  | **Close and empathetic relationship between volunteers and those supported** | |
|  | *"I think it ultimately comes down to this, or rather, the limit is, as far as we want it, because maintaining a very close emotional relationship with some is a matter of empathy or not..."*  **(Focus Group of Mental Health Volunteers 02, Volunteer 06)**  *"[...] they like us, we like them too, and we create an empathy, and we get along well , we don't even know why, but there are cases where I think it's necessary for us to have some knowledge of the person's illness, of what can suddenly happen to that person, because there are illnesses that can cause the person to change their personality, the way we talk to them, anything can trigger a reaction that's not very good."*  **(Focus Group of Mental Health Volunteers 02, Volunteer 05)** | *"[...] there's a phrase we like to use, which is 'We are the friend who arrives when everyone else leaves...'"*  **(Interview with Prison Volunteers, Volunteer 07)**  *"[...] it's a relationship of complicity; they end up getting to know us, and they are also free from any prejudice towards us. We are just people who are there to help, and that's quite positive."*  **(Interview with Prison Volunteers, Volunteer 10)**  *"[...] with them it's a close relationship; I make a point of addressing them by name and having them address me by my name as well, and they are just like us because that's exactly what they are."*  **(Interview with Prison Volunteers, Volunteer 15)** |
|  | **Greater receptivity towards volunteers compared to others** | |
|  | *"We don’t accept an opinion from a family member or a friend as easily; perhaps if it's someone who is not directly connected to us, they end up providing us with company, they end up talking a lot more, than perhaps…, there are things they tell the volunteer but not to their father..."*  **(Focus Group of Mental Health Volunteers 01, Volunteer 02)** | *"[...] they also saw us as outsiders with whom they could exchange ideas, vent a little, and talk about the difficulties they were facing..."*  **(Interview with Prison Volunteers, Volunteer 28)**  *"[...] there is a great need on their side to talk because we don't belong to the prison system, so they know that what they tell us stays with us."*  **(Interview with Prison Volunteers, Volunteer 35)** |
|  | **Personal growth of the volunteer** | |
|  | *"And I think volunteering really enriches us internally a lot."*  **(Focus Group of Mental Health Volunteers 01, Volunteer 03)**  *"[...] but ultimately, I found it rewarding because when they came to me, they wanted to know, I could see that they wanted to learn, even without being able to express themselves or speak, I could see it in their eyes that they wanted to touch what I was working with... it was indeed rewarding, and I would like to do it again and have these opportunities."*  **(Focus Group of Mental Health Volunteers 02, Volunteer 07)** | *"Now, on a human level, it certainly adds, of course, human knowledge, flexibility, a range of other things … I learn from it as a person, and I imagine that this always adds value to who I am as a professional in my field, as a daughter, sister, woman. I think it adds immense value to every aspect of my life, without a doubt, because I realise that my reality is not the only one... These experiences translate into real and distinctive skills for the volunteer and for any citizen."*  **(Interview with Prison Volunteers, Volunteer 25)** |
|  | **Relativization of volunteers’ problems** | |
|  | *“I also think that there are several volunteers who go through a phase of depression, and almost all of them say that it's very good to get out and help others, because perhaps they understand better the problems that others are going through, and for them, it's even a way of overcoming, of not thinking about their problems and focusing on others."*  **(Focus Group of Mental Health Volunteers 02, Volunteer 05)**  *"[...] it provided different solutions for a problem that I might have thought my solution was the only one. So it was more about becoming aware that in mental health, and in volunteering, truly becoming aware of the difficulties and problems that exist, and also the solutions."*  **(Focus Group of Mental Health Volunteers 02, Volunteer 06)** | *"In fact, going to the prison changes our perspective on how we look at other people... everyone... it changes the perspective with the inmate, changes the way we see them as men. They’re no longer just a men who stole or killed. It’s already a person, a much more complex human being, with good things..."*  **(Interview with Prison Volunteers, Volunteer 14)**  *"One of the main advantages is realising that our problems are not as serious when we hear the stories of some of the inmates. So, this relativisation of our problems, the deconstruction of this reality, the reduction of prejudices, the ability to listen, the emotional capacity..."*  **(Interview with Prison Volunteers, Volunteer 39)**  *"Benefit is waking me up to reality, drawing my attention to the context in which I live in is not the only one that exists, that there are other social, economic, spiritual, emotional, moral, and even religious contexts that are very different from mine. So it draws my attention to the fact that reality is more complex than the reality I experience every day."*  **(Interview with Prison Volunteers, Volunteer 16)** |
| **Differences** | **Learning about mental illness** | **Familiarization with the prison environment** |
|  | *"[...] I think I would have a completely different understanding of the human mind from the moment I knew something about the problems the human mind can suffer, wouldn't I?"*  **(Focus Group of Mental Health Volunteers 01, Volunteer 01)** | *"[...] it's a very special environment, very heavy, very tense, where there are many people the same age as my children, where there are people my age or older than me, and where there are people who are completely abandoned... and so, I didn't know about this before joining."*  **(Interview with Prison Volunteers, Volunteer 03)** |
| **Challenges faced by volunteers** | | |
| **Similarities** | **Observing significant results of volunteer support** | |
|  | *"[...] being able to reach the patient with a mental health condition and truly meet their basic needs, to help them understand, and also understanding if they saw my intentions, and that they, in the end, felt better..."*  **(Focus Group of Mental Health Volunteers 02, Volunteer 03)**  *"[...] if we see that the other person is not happy with our help, it's a bit frustrating for us. We are trying to help, and we don't see that we are helping; we are making the situation worse, and that is not very good for us."*  **(Focus Group of Mental Health Volunteers 01, Volunteer 04)** | *"I think it's very difficult to maintain their interest because they don't see the direct results... maintaining these people’s interest and really showing them the purpose we serve in their lives, showing our role, I think that's the big challenge."*  **(Interview with Prison Volunteers, Volunteer 30)**  *"The challenge is to be able to achieve positive results from situations that are initially negative."*  **(Interview with Prison Volunteers, Volunteer 33)** |
|  | **Deconstructing stigma and raising awareness in society** | |
|  | *"[...] interpreting the thoughts of people with mental illness is like treating them as if they were animals, as if they don't think 100% like us, right?"*  **(Focus Group of Mental Health Volunteers 01, Volunteer 03)**  "*My biggest challenge is even before anything else, making the relevant people to understand that mental health and the homeless are human beings who deserve all the dignity and not the kind of responses where people from community or therapeutic centers who ask them, 'either this or the street, no other option,' because the state has already spent too much money on you."*  **(Focus Group of Mental Health Volunteers 02, Volunteer 01)** | *"[...] another thing is society; they leave already with a label, and a criminal record doesn't disappear overnight. Nowadays, everyone wants to check the criminal record, and when it shows up, society doesn't accept them. And when it does accept them, we’ve seen some who were accepted, but later, when certain people in society find out that there’s a former prisoner, they make [their] life hell."*  **(Interview with Prison Volunteers, Volunteer 34)**  *"Usually, people imagine a scenario, everyone looking terrible, all degraded. And there, you find everything, there are many who are an example that in their day-to-day, they get dressed, they look neat, and there are people with degrees, lawyers, enforcement agents."*  **(Interview with Prison Volunteers, Volunteer 02)**  *"[...] I think many people go there, and they go with an almost a voyeuristic attitude, as if they were rare animals. They go to observe, but these people are perfectly normal, just like us. But they go there to conduct so many studies that sometimes they say, 'Oh my God, everyone seems to want to study us, as if we're animals in a cage, and everyone comes here to ask questions.'"*  **(Interview with Prison Volunteers, Volunteer 15)**  *"One thing I often say, which is that the rule of law is left at the prison gates... So, in prisons, there hasn’t been possible to change and advance humanisation because all parties represented in the Assembly of the Republic are afraid to move forward with any initiative because they believe they will be penalised in the elections [...] The biggest challenge is exactly this: changing public opinion because the political power is sensitive."*  **(Interview with Prison Volunteers, Volunteer 20)** |
| **Differences** | **Difficulties in dealing with people with mental illness** | **Confrontation with the prison environment as violent** |
|  | *"In other words, their world is different from ours, and perhaps it's not even physiological for us to understand what that person is thinking at that moment..."*  **(Focus Group of Mental Health Volunteers 01, Volunteer 01)**  *"When I dealt with people with mental disabilities, I was a bit confused about the way they approached me..."*  **(Focus Group of Mental Health Volunteers 02, Volunteer 07)**  *"Many times, I don't have a perception of whether the person is in their right mind..."*  **(Focus Group of Mental Health Volunteers 01, Volunteer 02)** | *"The adaptation to the prison environment itself, I think, is a very big challenge..."*  **(Interview with Prison Volunteers, Volunteer 37)**  *"I think everything in the prison context is a challenge, first because we are not psychologists, and second because we are dealing with people in circumstances completely different from our own."*  **(Interview with Prison Volunteers, Volunteer 33)** |
|  | **Shame and abandonment of people with mental illness** | **Focus on the crime committed by the inmate** |
|  | *"In the past, people were even ashamed when they had someone with disabilities at home […] They were ashamed, they would take them and put them in Conde Ferreira Hospital, and now it's not Conde Ferreira, it's Magalhães Lemos Hospital. And then they would even abandon them because they were ashamed that society knew they had mentally disabled people."*  **(Focus Group of Mental Health Volunteers 01, Volunteer 03)** | *"Much focus is placed on the crime the person committed and not on the person themselves."*  **(Interview with Prison Volunteers, Volunteer 16)**  *"The worst was this one; it's pretty clear. It was with a paedophile, you see, I couldn't get past it because I had a son, and they also had a son of the same age, and this paedophile would always ask us, 'And the boys?' and then they started showing us pictures of his godson who slept with him and gave him a gold chain, and that's when we almost ran away from him because I find paedophilia a horrible disease, a horrible perversion, children at risk..."*  **(Interview with Prison Volunteers, Volunteer 14)** |
